# Turning Platelets Off and On: Role of RhoGAPs and RhoGEFs in Platelet Activity

**DOI:** 10.3389/fcvm.2021.820945

**Published:** 2022-01-06

**Authors:** Shane P. Comer

**Affiliations:** ^1^ConwaySPHERE Research Group, UCD Conway Institute, University College Dublin, Dublin, Ireland; ^2^School of Biomolecular and Biomedical Science, University College Dublin, Dublin, Ireland

**Keywords:** platelets, signalling, Rho GTPases, GTPase activating protein (GAP), guanine nucleotide exchange factor (GEF)

## Abstract

Platelet cytoskeletal reorganisation is a critical component of platelet activation and thrombus formation in haemostasis. The Rho GTPases RhoA, Rac1 and Cdc42 are the primary drivers in the dynamic reorganisation process, leading to the development of filopodia and lamellipodia which dramatically increase platelet surface area upon activation. Rho GTPases cycle between their active (GTP-bound) and inactive (GDP-bound) states through tightly regulated processes, central to which are the guanine nucleotide exchange factors (GEFs) and GTPase-activating proteins (GAPs). GEFs catalyse the dissociation of GDP by inducing changes in the nucleotide binding site, facilitating GTP binding and activating Rho GTPases. By contrast, while all GTPases possess intrinsic hydrolysing activity, this reaction is extremely slow. Therefore, GAPs catalyse the hydrolysis of GTP to GDP, reverting Rho GTPases to their inactive state. Our current knowledge of these proteins is constantly being updated but there is considerably less known about the functionality of Rho GTPase specific GAPs and GEFs in platelets. In the present review, we discuss GAP and GEF proteins for Rho GTPases identified in platelets, their regulation, biological function and present a case for their further study in platelets.

## Introduction

**Figure 1:**
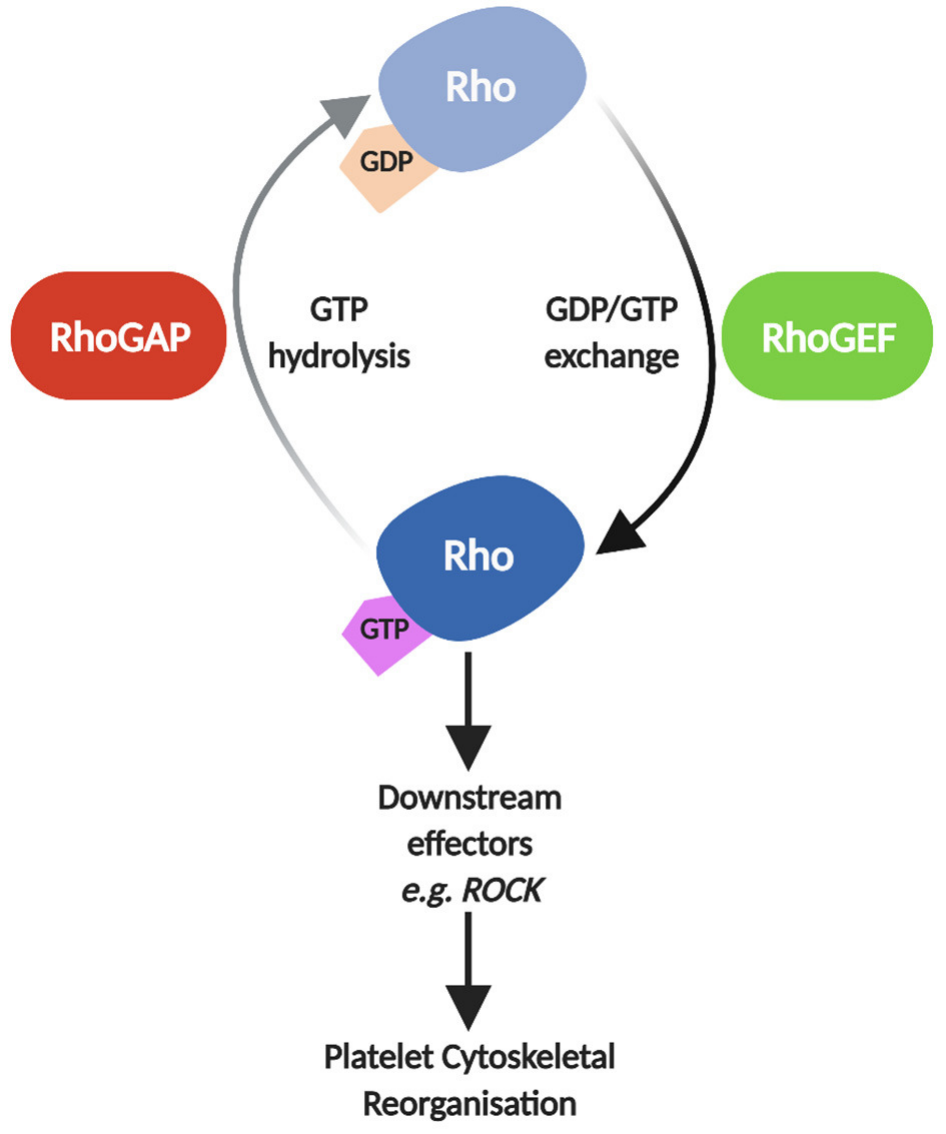
Schematic of Rho GTPase regulation by RhoGAPs and RhoGEFs. In their inactive state, Rho GTPases such as RhoA, Rac1 and Cdc42 are GDP-bound. GEFs facilitate the dissociation of GDP and binding of GTP, activating Rho GTPases allowing them to interact with downstream effectors such as Rho Kinase (ROCK), Filamin-A, and Wiskott-Aldrich syndrome protein (WASp) leading to reorganisation of the platelet cytoskeleton (2, 3). GAPs subsequently catalyse the hydrolysis of GTP to GDP, reverting the Rho GTPase to its inactive state, inhibiting Rho GTPase signalling. Image created using BioRender.

The molecular events that stimulate platelet adhesion, aggregation and thrombus formation are crucial to platelet function, both in haemostasis and thrombosis. It has been well documented that platelet regulation at a molecular level is a finely balanced system crosstalk between different signalling pathways, resulting in events such as phosphorylation, Ca^2+^ fluctuation, lipid modification and more (1). These processes are regulated and coordinated interdependently by small GTPases, allowing for the rapid alterations seen in the platelet cytoskeleton and overall platelet morphology upon activation (2–4). Approximately 8% of all proteins identified in platelets are small GTPases and their regulators (5). Despite the Rho family of small GTPases containing over 20 members, RhoA, Rac1 and Cdc42 are considered the primary drivers in the dynamic cytoskeletal reorganisation process, leading to the development of filopodia and lamellipodia (6). RhoA, Rac1 and Cdc42 have also been linked with other processes in platelet activation such as platelet granule release (2), clot retraction (7) and integrin activation via crosstalk with another small GTPase, Rap1 (8, 9). These proteins cycle between their inactive GDP-bound and active GTP-bound states and this cycling of activation is facilitated by regulatory proteins known as GTPase-activating proteins (GAPs–inhibitory) and guanine nucleotide exchange factors (GEFs – activatory) (10–12). GAPs catalyse the extremely slow intrinsic GTPase activity of Rho GTPases, terminating GTPase signalling (13), while GEFs facilitate the dissociation of GDP to GTP via their catalytic domains (10) (Figure 1).

While >70 RhoGAPs and >70 RhoGEFs (14, 15) have been identified in mammalian cells, platelets have been shown to express approximately 22 RhoGAPs and 27 RhoGEFs based on platelet proteomic analyses to date (Table 1) (5, 18, 20). However, few of these have been fully characterised in platelets, with detailed analysis on their role in platelet function severely lacking (14). Here, we provide an overview of the existing knowledge of identified RhoGAPs and RhoGEFs in platelets, including insights into their function, regulation and their role in platelet biology.

**Table 1:** Rho GTPase-activating proteins (RhoGAPs) and Rho guanine nucleotide exchange factors (RhoGEFs) expressed in human platelets.

		**Gene**	**Protein Name**	**Copy Number**
**RhoGAPs**		**ARHGAP1**	Rho GTPase-activating protein 1	9300
		ARHGAP18	Rho GTPase-activating protein 18	7100
		ARHGAP6	Rho GTPase-activating protein 6	4200
		HMHA1	Minor histocompatibility protein HA-1	3900
		BNIP2	BCL2/adenovirus E1B 19kDa interacting protein 2	3100
		ARAP1	Arf-GAP with Rho-GAP domain, ANK repeat and PH domain-containing protein 1	3100
		PIK3R1	Phosphatidylinositol 3-kinase regulatory subunit alpha	1900
		**ARHGAP17**	Rho GTPase-activating protein 17	1600
		INPP5B	Type II inositol 1,4,5-trisphosphate 5-phosphatase	1600
		**OPHN1**	Oligophrenin-1	1500
		**MYO9B**	Unconventional myosin-IXb	1400
		ARHGAP4	Rho GTPase-activating protein 4	1400
		GMIP	GEM-interacting protein	1200
		ARHGAP25	Rho GTPase-activating protein 25	1100
		ARHGAP10	Rho GTPase-activating protein 10	1100
		**ARHGAP21**	Rho GTPase-activating protein 21	890
		OCRL	Inositol polyphosphate 5-phosphatase OCRL-1	850
		ARHGAP15	Rho GTPase-activating protein 15	830
		BCR	Breakpoint cluster region protein	790
		RALBP1	RalA-binding protein 1	790
		**ARHGAP35**	Rho GTPase-activating protein 35	780
		SH3BP1	SH3 domain-binding protein 1	580
**RhoGEFs**	**Dbl Family**	**ARHGEF7**	Rho Guanine nucleotide-exchange factor 7	3100
		**VAV1**	Proto-oncogene vav	2100
		**VAV3**	Guanine nucleotide exchange factor VAV3	1800
		**ARHGEF6**	Rho Guanine nucleotide-exchange factor 6	1600
		FGD3	FYVE, RhoGEF and PH domain-containing protein 3	1600
		KALRN	Kalirin	1500
		**ARHGEF1**	Rho Guanine nucleotide-exchange factor 1	1400
		**ARHGEF2**	Rho Guanine nucleotide-exchange factor 2/GEF-H1	1400
		ITSN2	Intersectin-2	1200
		**ARHGEF12**	Rho Guanine nucleotide-exchange factor 12	920
		FGD4	RhoGEF and PH domain containing protein 4	840
		BCR	Breakpoint cluster region protein	790
		SOS1	Son of sevenless homologue 1	780
		OBSCN	Obscurin	660
		MCF2L	Guanine nucleotide exchange factor DBS	610
		AKAP13	A-kinase anchor protein 13	610
		**ARHGEF10**	Rho Guanine nucleotide-exchange factor 10	560
	**DOCK Family**	DOCK8	Dedicator of cytokinesis protein 8	1500
		DOCK5	Dedicator of cytokinesis protein 5	1500
		DOCK11	Dedicator of cytokinesis protein 11	1100
		DOCK10	Dedicator of cytokinesis protein 10	1000
		DOCK9	Dedicator of cytokinesis protein 9	760
		DOCK7	Dedicator of cytokinesis protein 7	710
		DOCK2	Dedicator of cytokinesis protein 2	670
		**DOCK1**	Dedicator of cytokinesis protein 1	TiO_2_ enrichment

*RhoGAPs (16) and RhoGEFs (17) known to be expressed in the human proteome were screened for protein expression against human platelet proteome datasets (5, 18, 19). Proteins are ranked according to their estimated copy numbers in platelets from Burkhart et al. (5). RhoGEFs are further subdivided into Dbl or DOCK families. Gene names highlighted in bold indicate proteins that have been characterised in platelets, beyond platelet proteome analyses*.

## RhoGAPs in Platelets

Disproportionately more (2-to-3 fold) GAPs are found in the human proteome compared to small GTPases and a number of theories have attempted to explain this difference (13). One theory is many GAPs exhibit specificity toward specific small GTPases. A caveat to this is many GAPs have been shown to exhibit some degree of regulatory ability to various small GTPases. A recent study by Müller et al. (12) has highlighted how RhoGAPs are more “promiscuous” than RhoGEFs regarding the number of GTPases which they can regulate. Another theory is the majority of GAPs are large, multi-domain molecules with functional roles in addition to their GAP activity, meaning they act as conduits of signal integration for various signalling pathways (13). Examples include the RhoGAP Bcr that possesses both GAP and GEF domains targeted to Rac1 and Cdc42, respectively (21). Another example is ARHGAP17 which expresses a PDZ binding domain, known to regulate multiple biological processes and signal transduction systems (22). Regulation of Rho GTPases by RhoGAPs in platelets remains poorly understood, with few of the approximately 22 RhoGAPs expressed in platelets having been thoroughly characterised. Platelets have previously served as a model system for investigations into RhoGAPs, a prime example being p50RhoGAP (ARHGAP1), a founding member of the RhoGAP family. First identified in platelets, p50RhoGAP was shown to have high specificity for Cdc42 (23) and subsequently RhoA and Rac1 in mammalian cell lines (21).

One of the first RhoA-specific GAP proteins characterised in platelets was p190RhoGAP (ARHGAP35), where its activity was stimulated during platelet activation via Src-family kinases (SFKs) inhibiting RhoA and thus facilitating platelet spreading (24). This is followed by inhibition of SFKs leading to an increase in RhoA-GTP levels promoting clot retraction (25). Activation of c-Src has also been suggested to mediate p190RhoGAP phosphorylation causing termination of RhoA signalling (24). Building on this, Flevaris et al. (25, 26) showed release of intracellular Ca^2+^ activated calpain proteases which cleave the integrin β3 subunit (activating c-Src), resulting in p190RhoGAP inhibition and RhoA reactivation to promote clot retraction.

Oligophrenin-1 (OPHN1) is one of the few RhoGAPs to have been studied extensively in platelets (27). Abnormal RhoA activation coupled with defective adhesion and lamellipodia formation was shown in platelets from OPHN1^−/−^ murine models (27). OPHN1 was also shown to localise to actin-rich regions in platelets where it exhibited regulatory roles in stress fibre, filopodia and lamellipodia formation, implicating it in the direct regulation of RhoA, Rac1 and Cdc42 (28). OPHN1 may have a role in platelet activation directly. OPHN1^−/−^ murine platelets exhibit abnormal RhoA hyperactivation and significant increases in thrombus formation both *ex-* and *in vivo* (27). These findings suggest aberrant regulation of Rho GTPases significantly impacts the ability of platelets to adhere and undergo classic shape change responses following stimulation of platelet activation (6).

ARHGAP17 (Nadrin) has been previously reported as a RhoGAP for Rac1 and Cdc42 in rat neuronal cells (29) and is described as a regulator of GAP activity through a mechanism of auto-inhibition (28). ARHGAP17 was shown to relocalise, similarly to OPHN1, to actin-rich regions within platelets, suggesting a cytoskeletal regulatory role for ARHGAP17 (30). Furthermore, it was shown to exhibit preferences for specific Rho GTPases dependent on the ARHGAP17 isoform being assessed (30). Nagy et al. (31) found PKA/PKG mediated phosphorylation of S702, which lies in a proline rich and intrinsically disordered region of the protein, facilitated CIP4 binding. CIP4 is a Rac1 effector involved in lamellipodia and filopodia formation. CIP4/ARHGAP17 dissociation occurred upon PKA activation and coincided with decreases in Rac1-GTP levels, however, a direct correlation between Rac1 inhibition and CIP4 binding could not be confirmed using a S702A mutant (31).

Myo9b (MyoIXb) is a member of the mammalian class IX myosins alongside Myo9a, which are unique amongst myosins due to the location of a GAP catalytic domain in the C-terminal tail region (32). Myo9b is found primarily in immune cells (33, 34) and has been shown to exhibit high RhoA specificity. The Myo9b rat homologue myr5 inhibits Cdc42 and Rac1, but at levels 100-fold (Cdc42) or 1000-fold (Rac1) greater than required for RhoA inhibition (35). It was also shown that Myo9b deficient prostate cancer cells and murine macrophages express higher levels of phosphorylated MLC, indicative of increased RhoA-GTP levels (36, 37). We previously found PKA/PKG phosphorylate Myo9b at S1354 in platelets enhancing Myo9b GAP activity leading to reduced RhoA-GTP levels. Myo9b phosphorylation, therefore, may contribute to local cyclic nucleotide-mediated control of RhoA and the actin/myosin cytoskeleton in platelets (38). Interestingly, in the same study we observed agonist-induced Myo9b phosphorylation suggestive of cAMP-independent PKA phosphorylation, previously proposed as an inhibitory feedback mechanism during thrombin- and collagen-induced platelet activation (38).

ARHGAP21 has previously been found to inhibit RhoA, RhoC (39) and Cdc42 in glioblastoma cell lines (40). Further, it has also been shown to play roles in various cytoskeletal processes such as cell migration (39) and adhesion (40) and stress fibre formation (41). Haploinsufficient (ARHGAP21^+/−^) mice were originally characterised as having decreased platelet counts and increased platelet volumes (42). Recently ARHGAP21 has been investigated as a key regulatory protein in megakaryocyte differentiation and platelet formation. ARHGAP21^+/−^ murine platelets exhibit enhanced thrombin-induced platelet aggregation, highlighting an increased thrombin sensitivity (39). ARHGAP21^+/−^ platelets also have increased P-selectin expression and increased levels of active RhoA and Cdc42 as well as enhanced thrombus formation (39).

The IQ-domain containing GAPs, IQGAP1 and IQGAP2, were reported to stabilise Rac1 and Cdc42 by binding to them directly [reviewed in (43)], however these GAPs do not express a true RhoGAP domain or exert any GTPase function (6). Interestingly, Bahou et al. (44) showed thrombin, but not collagen treatment induced the relocalisation of IQGAPs to the platelet cytoskeleton, specifically the cytoskeleton of filopodia. This suggests IQGAPs are activated downstream of the GPCRs PAR1/PAR4 but not GPVI.

The above discussed RhoGAPs are currently the only ones to have been investigated in detail in platelets. Aside from these identified RhoGAPs, there are potentially many more expressed in platelets, as platelet proteomic and transcriptomic studies have shown (5, 45).

## RhoGEFs in Platelets

In contrast to RhoGAPs, there are slightly more studies which characterise functions and roles of specific RhoGEFs in platelets. Humans express approximately 81 RhoGEF proteins, exceeding the number of potential target small GTPases by a ratio of almost 4:1 (15). These RhoGEFs are classed into two distinct families: the Dbl (~70 members) and Dock (~11 members) families. Diffuse B-cell lymphoma (Dbl) GEFs are characterised their classic tandem domain structure of a Dbl homology (DH) catalytic GEF domain, and the pleckstrin homology (PH) domain. The PH domain primarily stabilises the DH domain allowing it to catalyse the GDP-to-GTP switch (46), amongst other functions such as phosphoinositide binding (47). Each member of the Dbl family contains other distinct domains and motifs which facilitate their interaction with other proteins, cellular structures and make them candidates for various post-translational modifications (17). Members of the dedicator of cytokinesis (Dock) family are noted for the catalytic Dock homology region 2 (DHR2) GEF domain and the DHR1 domain, located C-terminally to DHR2 and involved in membrane localisation (48). Dbl family GEFs are known to exhibit varied specificities to Rho GTPases, whereas the Dock family are thought to be restricted to Rac/Cdc42 regulation [reviewed in (49–51)]. Although, recent work has shown reduced RhoA activity due to downregulation of Dock1 in triple negative breast cancer epithelial cells (52). Further, a weak interaction of Dock 10 with RhoF and RhoG *in vitro* has also been reported (53). Goggs et al. (3, 54) using a RhoG-GTPγS pull-down proteomic approach found platelets express Dock1, Dock5 and Dock10 although their role in platelet function, indeed the role of the Dock family of GEFs, remains poorly understood. RhoGEFs of the Dbl family are the focus of the present review.

One of the best characterised RhoGEFs in platelets is the RhoA-specific ARHGEF1 (p115RhoGEF) (2). Downstream of GPCR stimulation, G_α13_ was shown to bind ARHGEF1 directly through the G_α13_ switch region 1 (SRI) thus promoting ARHGEF1 mediated RhoA activation (55). Furthermore, a recent study using ARHGEF1 knockout mice found significantly prolonged thrombus occlusion and increased tail bleeding times. These mice also displayed significantly attenuated platelet aggregation, α_IIb_β_3_ activation and granule release in response to a variety of platelet agonists (56). Interestingly, ARHGEF1 was reported to act as a RhoGAP for G_α13_ via its regulator of G-protein signalling (RGS) domain, as well as functioning as a RhoGEF for RhoA. This potentially reveals a system of negative feedback on RhoA-GTP signalling; ARHGEF1 acting as an intermediary in G_α13_ regulation of RhoA (57).

GEF-H1 (ARHGEF2) was initially characterised as a Dbl RhoGEF that integrated microtubule dynamics to cell contractility (58). Gao et al. (59) found decreases in RhoA-GTP levels during murine megakaryocyte endomitosis corresponded to downregulation of GEF-H1 at mRNA and protein levels. Exogenous expression of GEF-H1 was also found to induce low ploidy in developing megakaryocytes (59). Aslan et al. (60) subsequently reported GEF-H1 as a substrate for p21 activated kinases (PAKs) and associates with Rac1-GTP in response to thrombin. We previously reported PKA/PKG phosphorylation of GEF-H1 at S886 increases 14-3-3β interaction, inactivating GEF-H1 and decreasing RhoA-GTP (38). In the same study we found nocodazole-induced disruption of platelet microtubules increases RhoA-GTP levels but does not affect S886 phosphorylation or RhoA inhibition. This is noteworthy as GEF-H1 is the only GEF reported to localise at cell microtubules (61), giving more insight into platelet cytoskeletal dynamics.

ARHGEF6 (αPIX, Cool-2), a Rac specific RhoGEF, was first identified in platelets (excluding proteomic studies) in 2013. Aslan et al. (60) successfully used Rac1-GTPγS to isolate ARHGEF6 (and ARHGEF7) from thrombin-activated human platelets. The SH3 domain of ARHGEF6 was previously shown to interact with PAK1-3 *in vitro* (62), well known downstream effectors of Rac and Cdc42 in human platelets (63). Nagy et al. (31) discovered ARHGEF6 is constitutively associated with GIT1 (ArfGAP1) in platelets. GIT1 also contains a GAP domain specific for the small GTPase Arf6 meaning GIT1 could contribute to the suppression of Arf6-GTP levels occurring during platelet activation (64). In the same study, the authors showed PKA/PKG-mediated phosphorylation of S684 promoted 14-3-3 binding to ARHGEF6, reducing Rac1-GTP levels (31).

The RhoA-specific ARHGEF10 was shown by Matushita et al. (65) to be associated with increased risk of atherothrombotic stroke via a specific single-nucleotide polymorphism. Murine ARHGEF10^−/−^ platelets exhibited reduced aggregation in response to various agonists and protected mice from thrombus formation (66). Lu et al. (66) reported ARHGEF10^−/−^ platelets show decreased ROCK phosphorylation, representative of reduced G_α13_-mediated RhoA activation. This study highlights a key role of ARHGEF10 in RhoA regulation and normal platelet function.

ARHGEF12 (LARG) is member of the Dbl family of RhoGEFs which has been well characterised in platelets. Williams et al. (67) found ARHGEF12^−/−^ murine platelets were unaffected during thrombopoiesis but exhibited a significant reduction in aggregation and dense granule secretion in response to U46619 (TXA_2_ synthetic analogue) and PAR receptor stimulation but not ADP. Platelet spreading and adhesion in response to fibrinogen or collagen-related peptide (CRP) were also unaffected (67). This suggests ARHGEF12 is stimulated downstream of G_α13_ coupled receptors. However, the authors found ARHGEF12 deletion only affected basal RhoA activity not agonist-induced activity. Therefore, ARHGEF12 may be responsible for RhoA activity in resting platelets only (67). In contrast, Zou et al. (68) reported ARHGEF12 was necessary for RhoA activation in platelets. MLC phosphorylation by ROCK (downstream of RhoA-GTP) was abolished, albeit in a global ARHGEF12 knockout mouse model. Zou et al. (68) also noted differences in platelet agonist treatment times and dosages as potentially being responsible for the observed increase in ARHGEF12-mediated RhoA activation. Despite the differences between these studies, both confirmed ARHGEF12 functions downstream of G_α13_ and is necessary for normal platelet function, however, the impact on RhoA activation warrants further confirmatory investigations.

The Vav subgroup of Dbl RhoGEFs, comprised of Vav1-3 are well documented in platelets. This subgroup have been described as exhibiting GEF function toward Rho, Rac and Cdc42 but have a particular affinity for Rac proteins (2, 15). Vav activation in platelets was reported downstream of thrombin and collagen stimulation. Thrombin-stimulated PAR signalling and collagen interaction with α_2_β_1_ induced tyrosine kinase mediated phosphorylation of Vav within 15 seconds (69). Interestingly, ADP and U46619 did not induce any detectable tyrosine kinase-mediated phosphorylation of Vav, suggesting Vav phosphorylation and activation was platelet agonist specific (69). Pearse et al. (70) reported a minor role for Vav1 in platelet function, specifically during the later stages of thrombin- or CRP-induced platelet aggregation. Later studies by Pearce et al. (70, 71) assessed the function of Vav3 in conjunction with Vav1 and showed both were required for platelet spreading through PLCγ regulation by α_IIb_β_3_ (72). Vav1/3 have been shown to form a complex with another Rac-specific GEF, P-Rex1, regulating signalling events during thromboinflammation (73). P-Rex1^−/−^ platelets have attenuated aggregation and secretion in response to collagen and GPCR agonists (74) but do not have impeded platelet spreading (75). RhoGEFs such as TIAM1 (76), Sos1 (77) and TRIO (3) have all been proposed to be expressed in platelets, however, their functional relevance still remains unclear.

## Conclusions

In recent years, it has become apparent that activatory and inhibitory platelet signalling pathways do not function independently of one another (1). There is considerable overlap and crosstalk between both systems which act together to regulate platelet function. Interestingly, this crosstalk is very often focused on the same proteins i.e., proteins that can be differentially regulated by both platelet activators and inhibitors (1). Recent work has shown how RhoGAPs and RhoGEFs function collectively with systems-level behaviour to manage Rho activity in specific cellular regions. Müller et al. (12) established a regulator-centric model of Rho regulation whereby the regulators (RhoGAPs and RhoGEFs) supply spatiotemporal information based on their location on specific cellular structures. This suggests RhoGAPs and RhoGEFs are primed to respond to localised regulation, allowing for expedient modification to various stimuli. We see evidence for this in RhoA regulation. Graessl et al. (78) reported Myo9b and GEF-H1 form a network with RhoA and actin filaments, generating dynamic patterns of subcellular contractility in adherent U2OS cells.

The functional diversity of Rho GTPases downstream of platelet signalling pathways highlights the need for regulators of these molecular mechanisms. RhoGAPs and RhoGEFs are centrally positioned as conduits for various interweaving activatory and inhibitory signalling pathways in platelets, contributing to effective haemostasis (1, 79). Rho GTPases also play roles in various pathologies (14) and targeting Rho GTPase pathways has been a focus of pharmacological advancements, particularly in potential cancer therapies (80), a recent success being new direct RasG12 inhibitors against cancer (81). However, very few therapies that target Rho GTPase signalling have been developed beyond clinical pretrials [reviewed in (82)].

Much effort has been spent on developing compounds which inhibit GDP/GTP exchange which suggests strategic targeting of RhoGAPs and RhoGEFs could lead to greater selectivity in targeted treatments. Examples of this include development of ARHGEF12 inhibitors (83) and the Rac1/Vav2 interaction inhibitor EHop-016 (84). Research targeting RhoGAPs is limited, but the goal is to enhance GTPase activity. There is limited evidence for targeting the Chimaerin C1 domain of the Rac-specific Chimaerin RhoGAP family (85). C1-binding small molecules may enhance chimaerin GAP activity (86) although potential for off-target effects is considerable as many proteins express C1 domains.

Presently, we are only beginning to understand the multifaceted roles RhoGAPs and RhoGEFs play in Rho GTPase regulation in platelets. Platelet proteomic studies (5, 18, 20) have provided a clearer picture of how many RhoGAPs and RhoGEFs are expressed in platelets, but characterisation studies focused on their specific function in platelets will be the key to providing new insights. Future work should investigate pharmacological inhibition/activation of specific RhoGAPs and RhoGEFs in platelets, with the aim of identifying targets which can function as anti-platelet therapies while preserving haemostatic function.

## Author Contributions

SC conceptualised, researched, and wrote the article.

## Conflict of Interest

SC is the Sanofi S.A. Newman Fellow in Haematology. Sanofi S.A. had no input into the preparation of the manuscript or decision to publish.

## Publisher's Note

All claims expressed in this article are solely those of the authors and do not necessarily represent those of their affiliated organizations, or those of the publisher, the editors and the reviewers. Any product that may be evaluated in this article, or claim that may be made by its manufacturer, is not guaranteed or endorsed by the publisher.

## Glossary

A: alanine
ADP: adenosine diphosphate
Arf: ADP-ribosylation factor
ARHGAP: Rho GTPase-activating protein ARHGEF, Rho guanine nucleotide exchange factor
Bcr: breakpoint cluster region protein
Ca^2+^: calcium ion
CalDAG-GEFI: Calcium and DAG-regulated guanine nucleotide exchange factor I
cAMP: cyclic adenosine monophosphate
Cdc42: cell division control protein 42
CIP4: CDC42-interacting protein 4
CRP: collagen-related peptide
C3G: RapGEF1
Dbl: diffuse B-cell lymphoma
DH: Dbl homology
DHR: dock homology region
DOCK: dedicator of cytokinesis
GAP: GTPase activating protein
GDP: guanine diphosphate
GEF: guanine nucleotide exchange factor
GIT1: ArfGAP1
GP: glycoprotein
GPCR: g-protein coupled receptor
GTP: guanine triphosphate
IQGAP: IQ calmodulin-binding motif containing GTPase activating protein
LARG: leukaemia-associated Rho GEF (ARHGEF12)
MLC: myosin light chain
Myo9b: unconventional myosin IXb
OPHN1: Oligophrenin-1
PAK: p21-activating kinase
PAR: protease activated receptor
PDE3A: phosphodiesterase 3A
PDZ: post synaptic density protein (PSD95), Drosophila disc large tumour suppressor (Dlg1), and zonula occludens-1 protein (zo-1)
PH: pleckstrin homology
PKA: protein kinase A
PKG: protein kinase G
PLCγ: phospholipase C gamma
P-Rex1: PI(3,4,5)P3-dependent Rac exchanger 1 protein
Rac1: Ras-related C3 botulinum toxin substrate 1
Rap1: Ras-related protein 1
RASA3: Ras GTPase-activating protein 3
RGS: regulator of g-protein signalling
RhoA: Ras homology family member A
Rho GTPase: Ras homology family GTPase
ROCK: Rho kinase
S: serine
SFK: Src family kinase
SH3: Src homology 3 domain
Sos1: son of sevenless homologue 1
Src: Proto-oncogene tyrosine-protein kinase Src
SRI: switch region1
TIAM1: T-lymphoma invasion and metastasis-inducing protein 1
TRIO: triple functional domain containing protein
TXA_2_: thromboxane A_2_
Vav: vav guanine nucleotide exchange factor
WASP: Wiskott–Aldrich syndrome protein.
